# T246. DECREASING AGGRESSIVE BEHAVIOR IN PATIENTS WITH COGNITIVE IMPAIRMENTS BY TRAINING PSYCHIATRIC STAFF IN INTERACTIVE SKILLS

**DOI:** 10.1093/schbul/sby016.522

**Published:** 2018-04-01

**Authors:** Daniel Abrams, Anneli Goulding, Margda Waern, Nils Sjöström

**Affiliations:** 1 University of Gothenburg; 2 University of Gothenburg, Sahlgrenska University Hospital

## Abstract

**Background:**

Preventive measures to decrease aggressive incidents in psychiatric care range from friendly responses to advanced de-escalation techniques.

But interventions have not often been systematically evaluated and often have different emphasis. There is also large variation in the outcome measurements used.

A method that has been used in Sweden is an interactive training approach, which aims to establish and maintain calmness and security for patients with cognitive impairments. Experiences from Gothenburg indicate decreased levels of coercive measures after training staff and providing supervision. The in-patient-unit where such training and application has been carried out most consistently, won a national award in 2016 for having no coercive measures taken in six months, despite 90 percent of the patients receiving compulsory care.

The intervention is a well defined 3-day-course, with two trainers and twelve participants. The main part of the course is devoted to the role playing of conflict situations with patients, based on the participants’ own experiences and examples. Visual analysis tools are used to make the role plays into learning situations.

**Aim:**

We describe here the study protocol for a planned project that will test the Interactive Training approach in four regional hospitals. In addition, group interviews will be applied to increase understanding of staff experiences, as well as the evaluation of the implementation process.

**Methods:**

Planned sub-studies:

1. Staff’s experience of using interactive methods will be analyzed through focus-groups; four group interviews with 5 people in each group. (Assisting nurses and nurses working full-time, who have been educated in interactive conflict-handling and worked according to the method for at least one year).

2. Intervention study. The staff at the psychiatric departments of four different hospitals will receive training in interactive conflict handling, and after the course, supervision. The purpose is to compare the number of aggressive events before and after the intervention.

The instruments that will be used for measurement of the effect are the Staff Observation Aggression Scale - revised (primary outcome), the Social Dysfunction Aggression Scale and the Clinical Global Impression - Severity Scale.

We will also document the type of care (voluntary or compulsory), the number of psychiatric hospital beds, the number of inpatient patients, the number of staff employed, if the patient was affected by alcohol or illegal drugs and several other variables. Diagnoses will be retrieved from patient records.

3. Evaluation of implementation. The purpose is to analyze the implementation of the intervention at four hospitals. Group interviews will be conducted and the data will be analyzed qualitatively by using Normal Process Theory (NPT) as a framework. NPT is an action research perspective that focuses on what actors actually do and discerns between, implementation, embedding integration as different levels of change.

**Results:**

Data collection for the first sub-study will be completed in June 2018 and results from the second and third are anticipated to be available by March 2019 and December 2019, respectively.

**Discussion:**

Possible methodological problems are that data from focus-groups may not be possible to generalize. However, qualitative data may capture experiences that shed light on the psychological working-mechanisms of the intervention.

The intervention study is expected to generate rich data, where essential variables are controlled for, for example organizational features, distribution of diagnoses and severity of symptoms. However, in a complex organization, it may not be possible to control for all variables that might explain variations in outcome.

